# SWI/SNF complex alterations as a biomarker of immunotherapy efficacy in pancreatic cancer

**DOI:** 10.1172/jci.insight.150453

**Published:** 2021-09-22

**Authors:** Gregory P. Botta, Shumei Kato, Hitendra Patel, Paul Fanta, Suzanna Lee, Ryosuke Okamura, Razelle Kurzrock

**Affiliations:** 1 Center for Personalized Cancer Therapy, Department of Medicine, and; 2 Division of Hematology/Oncology, Department of Medicine, UCSD, La Jolla, California, USA.; 3 Department of Surgery, Kyoto University Hospital, Kyoto, Japan.

**Keywords:** Oncology, Immunotherapy

## Abstract

**BACKGROUND:**

Immune checkpoint inhibitors (ICIs) fail to demonstrate efficacy in pancreatic cancer. Recently, genomic biomarkers have been associated with response to ICIs: microsatellite instability high (MSI-H) and tumor mutation burden (TMB) > 10 mutations/Mb. Alterations in Switch/Sucrose Nonfermentable (SWI/SNF) chromatin remodeling genes may predispose to improved outcomes with immunotherapy. The current study examined a possible role for SWI/SNF complex abnormalities in pancreatic cancer responsiveness to ICIs.

**METHODS:**

A database of 6831 cancer patients that had undergone next-generation sequencing (NGS) was filtered for advanced pancreatic cancer, SWI/SNF alterations, and outcomes depending on immunotherapy treatment.

**RESULTS:**

Nine patients had metastatic pancreatic adenocarcinoma harboring SWI/SNF chromatin remodeling gene alterations and had received ICIs: 7 had an ARID1A alteration (77%); 2, ARID1B (22%); 3, SMARCA4 (33%); 1, SMARCB1 (11%); and 1, PBRM1 (11%). Three patients possessed more than 1 SWI/SNF complex alteration. Only 3 tumors were microsatellite unstable. Eight of 9 patients (89%) achieved an objective response, including a complete remission, with the 2 longest responses ongoing at 33+ and 36+ months. Median progression-free and overall survival was 9 and 15 months, respectively. Responses occurred even in the presence of microsatellite stability, low TMB, and/or low PD-L1 expression.

**CONCLUSION:**

A small subset of patients with pancreatic cancer have genomic alterations in SWI/SNF chromatin remodeling components and appear to be responsive to ICIs, suggesting the need for prospective trials.

**TRIAL REGISTRATION:**

ClinicalTrials.gov, NCT02478931.

**FUNDING:**

Joan and Irwin Jacobs Fund, NIH P30 CA023100 (RK) and LRP KYGF9753 (GPB), the Gershenson, Duarte, and anonymous patient families (GPB).

## Introduction

Pancreatic adenocarcinoma is currently the third leading cause of cancer-related death in the United States, and over 90% of afflicted patients die from this disease (1). The only potential cure for pancreatic cancer is surgical resection; however, only approximately 10%–15% of patients have nonmetastatic, resectable disease at diagnosis. Even after pancreaticoduodenectomy, the 5-year survival is ~10% for lymph node–positive disease and slightly improved (~30%) with lymph node–negative disease (2). For the majority of patients presenting with metastatic disease, the 2-year survival is 6.4%, with a 5-year survival of only 2.5% (3).

Chemotherapy in the form of FOLFIRINOX (5-fluoruracil, leucovorin, irinotecan, oxaliplatin) or gemcitabine and nab-paclitaxel are the backbone of modern metastatic pancreatic cancer therapy (4, 5). Generally, each regimen is chosen on the basis of the patient’s performance status and ability to cope with the specific side effect profiles of the 2 regimens. Since many patients with metastatic pancreatic cancer suffer from multiple comorbidities that develop with their disease, they are often subjected to treatment interruptions, dose reductions, and palliative single-agent therapies.

Meanwhile, the use of other targeted compounds has proven futile in pancreatic cancer, perhaps because they have been generally applied without biomarker selection (6). Specifically, small molecule inhibitors, tumor microenvironment regulators, and immunotherapy have all failed in late-phase pancreatic cancer clinical trials. While limited success (i.e., an increase of progression-free survival [PFS] of about 2 weeks) was described with the epidermal growth factor receptor (EGFR) tyrosine kinase inhibitor erlotinib, it is rarely used because of the paltry improvement in outcome and side effect profile (7). However, gene profiling of pancreatic cancer has uncovered that ~5% of patients with metastatic pancreatic cancer harbor a germline *BRCA* mutation and respond favorably to platinum-based chemotherapies, followed by maintenance small-molecule poly(ADP-ribose) polymerase (PARP) inhibitors (8).

A modality of interest across all cancers includes immune checkpoint inhibitors (ICIs). To date, 6 anti–programmed death receptor-1/programmed death receptor-ligand 1 (anti–PD-1/PD-L1) agents and 1 anti–cytotoxic T-lymphocyte-associated protein 4 (anti–CTLA-4) agent are approved for a variety of cancers (9). Recently, approval of pembrolizumab has been extended to include microsatellite instability–high (MSI-H)/deficient mismatch repair (dMMR) and tumor mutational burden (TMB) > 10 mutations per megabase (mut/Mb) cancers regardless of tissue of origin (10–12). Even so, investigators have questioned the threshold of TMB of 10 mut/Mb, especially for cancers such as pancreatic, in which few immunotherapy responses have been documented. Furthermore, only ~2% of pancreatic cancer patients have MSI-H, and they are usually within clusters of Lynch syndrome families (13, 14). As such, over 98% of pancreatic cancers are deemed “cold,” having an uninflamed microenvironment incapable of spurring an immune response to checkpoint inhibition (15–18). Indeed, trial after trial has shown that immunotherapeutics have limited efficacy in advanced and metastatic pancreatic cancer when evaluating unselected patients, providing little improvement in survival beyond traditional chemotherapy (15, 19–24).

**Figure 1:**
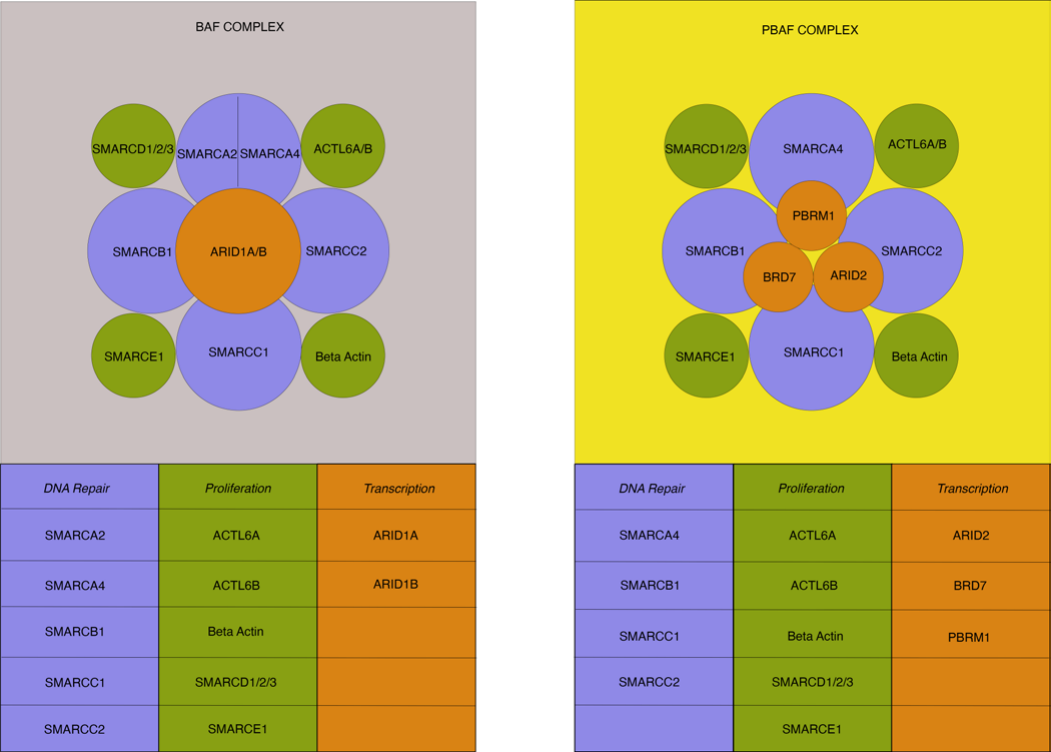
SWI/SNF complexes. The human SWI/SNF complex mediates chromatin remodeling and is composed of 2 subclasses: BAF and PBAF structures. Multiple subunits (between 8 and 14) comprise each structure with core homology between DNA repair (light blue) and proliferation (green) subunits. Transcriptional subunits (orange) differentiate the 2 classes. The BAF complex has either SMARCA2 or SMARCA4 as a DNA repair subunit — but not both. DNA repair subunits are implicated in the nucleotide excision and double-strand break repair. Each subunit is identified by its specific gene name, which is associated with the translated protein of the complex. BAF, BRG1 (SMARCA4)- or BRM (SMARCA2)-associated factors; PBAF, Polybromo-associated BAF.

The SWItch/Sucrose Nonfermentable (SWI/SNF) complex is a subfamily of adenosine triphosphate–dependent (ATP-dependent) chromatin remodeling proteins that alter nucleosome topology and DNA access, ultimately regulating gene transcription (25) (Figure 1). Although gene alterations in individual complex members are common during carcinogenesis, their roles in phenotype and exploitability for drug targeting are not well delineated. Pancreatic cancer has been found to have SWI/SNF complex alterations between 2.5% and ~18% of the time by large-scale sequencing (26). Inactivating mutations, deletions, substitution or frameshift alterations, insertions, allelic loss, rearrangement, or truncation in several SWI/SNF genes — including but not limited to *ARID1A, SMARCA4*, and *PBRM1* — have been implicated in responsiveness to ICIs in a variety of cancers (27–34). This is perhaps due to the fact that lower expression of SWI/SNF complex members is associated with higher CD8^+^ cytotoxic T cell activity in human cancers, and their inactivation in human cancer cell lines sensitizes tumors to T cell–mediated cytotoxicity (27, 33, 35–37). To date, however, there has been no analysis as to whether this subpopulation of pancreatic cancer is responsive to immunotherapy compared with clinical trials evaluating all comers.

Herein, we examined patients with refractory metastatic pancreatic adenocarcinoma who showed alterations in 1 or more SWI/SNF complex chromatin remodeling genes and who received an anti–PD-L1 or anti–PD-1 agent. Our results suggest that patients harboring SWI/SNF-altered pancreatic cancer can respond to ICI.

## Results

### Patient demographics.

**Figure 2:**
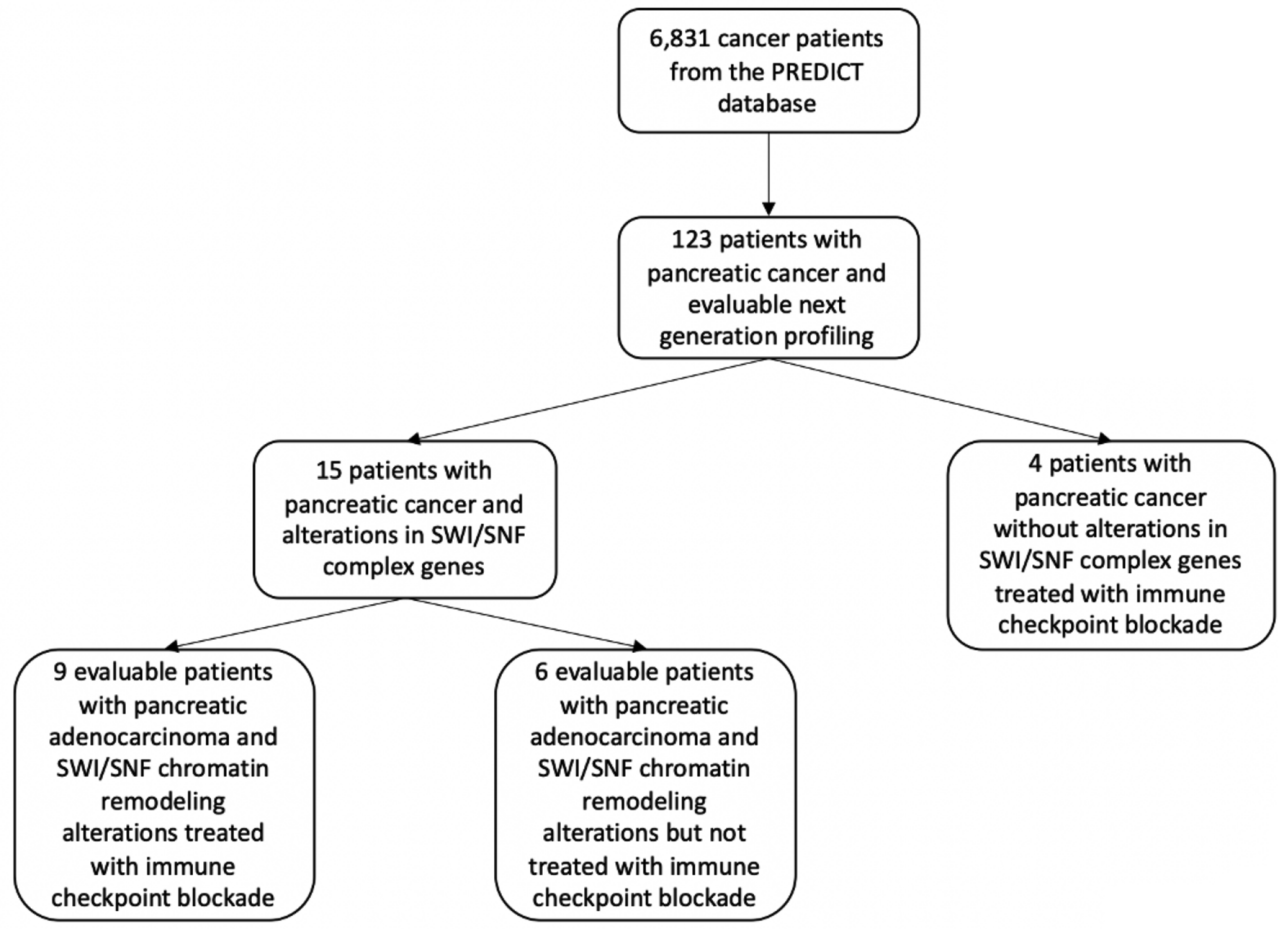
CONSORT diagram. Pancreatic cancer patients with the aforementioned tumor and treatment characteristics were extracted from the Profile-Related Evidence Determining Individual Cancer Therapy (PREDICT) database at the UCSD Center for Personalized Cancer Therapy. Nine total evaluable patients were identified with SWI/SNF alterations and who received immunotherapy. Six patients with SWI/SNF alterations who did not receive immunotherapy and 4 pancreatic cancer patients without SWI/SNF alterations who did not receive immunotherapy were used as comparator arms.

There were 6831 eligible cancer patients in the Profile-Related Evidence Determining Individual Cancer Therapy (PREDICT) database (NCT02478931; ClinicalTrials.gov). Of these individuals, 293 (4%) patients had metastatic pancreatic cancer, and of these, 123 (42%) had clinical-grade (tissue DNA and/or blood circulating tumor DNA [ctDNA]) NGS performed (Figure 2). These 123 pancreatic cancer patients were further substratified to those 15 persons (12%) with definable, SWI/SNF complex alterations found by next-generation sequencing (NGS). Of this cohort, 9 patients had SWI/SNF complex alterations and were evaluable for checkpoint inhibitor immunotherapy. Six patients with metastatic pancreatic cancer and SWI/SNF complex alterations not treated with immunotherapy were also analyzed.

Of the 9 evaluable immunotherapy-treated, SWI/SNF-altered pancreatic cancer patients, 3 were men (33%), 6 were women (67%), and the median age was 66.5 years (range, 47–79 years) (Supplemental Table 3; supplemental material available online with this article; https://doi.org/10.1172/jci.insight.150453DS1). The patients came from a variety of race and ethnicities: Hispanic/Latino, African American, White, Asian, and Pacific Islander. The median follow-up from the date of starting ICI was 9 months, and the mean was 12.7 months (range, 1–36 months). Of the 6 evaluable nonimmunotherapy-treated*,* SWI/SNF-altered patients, 3 were men (50%), 3 were women (50%), and the median age was 64.5 years (range, 31–76 years) (Table 1).

### NGS and immunotherapeutic intervention.

Altogether, in patients with available data, 3 of 11 SWI/SNF-altered tumors had TMB ≥ 10 mut/Mb, and 3 of 12 patients had tumors that were microsatellite unstable (Tables 1 and 2); 2 of 13 patients had intermediate expression of PD-L1 by IHC.

**Figure 3:**
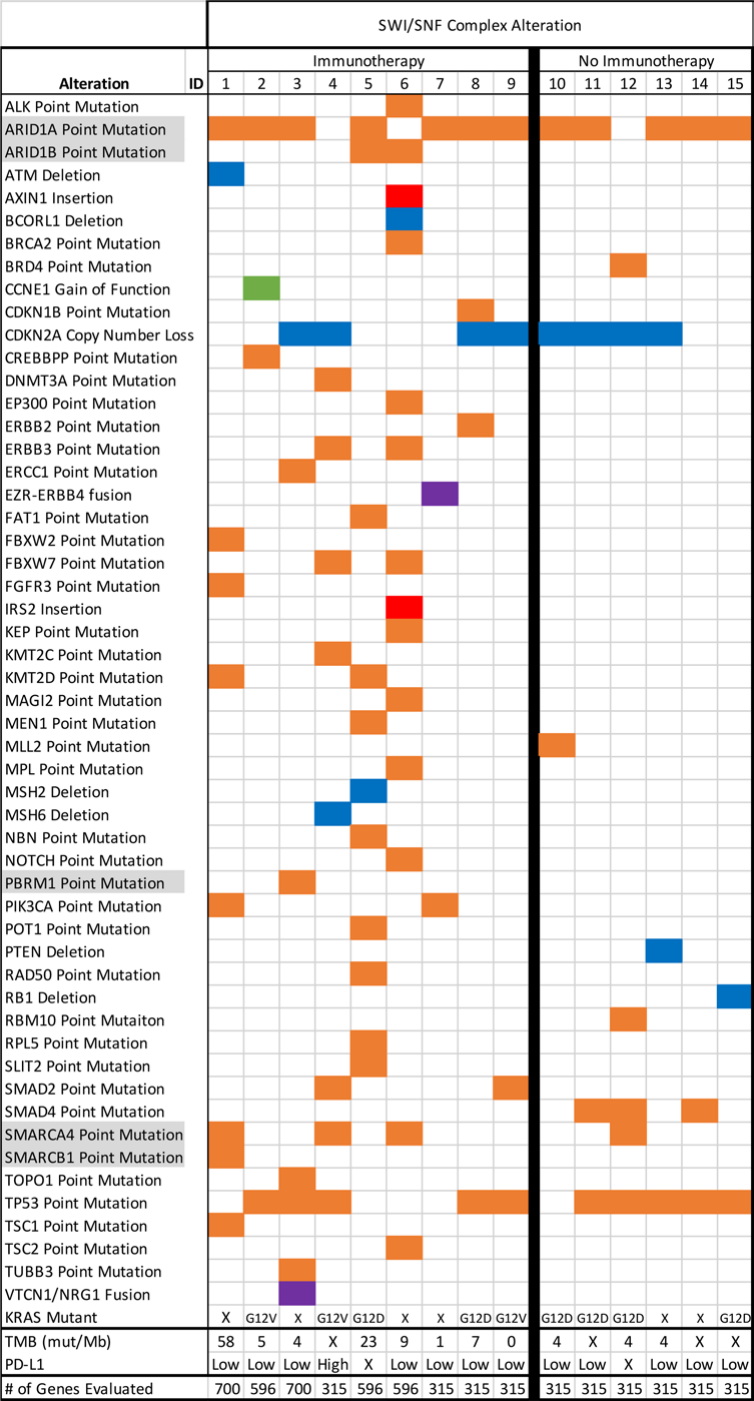
Molecular characteristics of pancreatic cancer patients with SWI/SNF alterations with and without immunotherapy. Molecular analysis of each patient (ID). Orange boxes represent point mutations, blue boxes are deletions, red boxes are insertions, green boxes are gain-of-function (GOF), and purple boxes are fusions. *KRAS* mutations are described as none (X) or the specified point mutation. Tumor mutation burden (TMB) is described by mutations per megabase (mut/Mb) or none (X). PD-L1 is stratified on the scale: low (0%–1%), intermediate (2%–49%), high (50%+), or none (X) using the Dako 22C3 pharmDx qualitative immunohistochemical assay of tumor cells (38). The total number of genes analyzed per patient tumor sample is specified.

On tissue NGS of immunotherapy-treated, SWI/SNF-altered pancreatic cancer patients, 7 patients harbored an *ARID1A* alteration (77%); 2 harbored an *ARID1B* mutation (22%); 3 harbored a *SMARCA4* mutation (33%); 1 harbored a *SMARCB1* mutation (11%); and 1 harbored a *PBRM1* mutation (11%). Three patients possessed more than 1 SWI/SNF complex alteration (ID #1: *ARID1A*, *SMARCA4*, *SMARCB1*; ID #5: *ARID1A*, *ARID1B*; ID #6: *ARID1B*, *SMARCA4*). Of note, 4 patients (44%) lacked a *KRAS* mutation (details of both tissue and blood NGS are in Supplemental Table 3 and Figure 3). The nonimmunotherapy-treated SWI/SNF-altered pancreatic cancer patients harbored 5 *ARID1A* alterations (83%) and 1 *SMARCA4* mutation (17%); none possessed more than 1 SWI/SNF complex alteration. Interestingly, 2 patients of 5 evaluated patients (40%) also lacked a *KRAS* mutation (Table 1 and Figure 3).

TMB was evaluated in 8 immunotherapy-treated SWI/SNF-altered patients (Supplemental Table 3). The median of the highest TMB in each patient, represented by mut/Mb was 7.5 (range, 0–58). Three patients had TMB evaluated more than once, and the TMBs differed (8 versus 3.3, 7 versus 2, and 11 versus 8.3; all mut/Mb). In each of these patients, the TMB was evaluated by different laboratories and from different biopsy specimens. The majority of patients had proficient MMR proteins (*n* = 6; 67%), but 3 patients had deficient MMR proteins (33%). Six patients (67%) had PD-L1–low scores (0%–1%), 2 (22%) were intermediate (2%–49%), and 1 was not determined (ID #5) (all IHC) (38). Seven total pancreatic cancer patients were evaluated for tumor-infiltrating lymphocytes (TILs), with 3 immunotherapy-treated patients having moderately to highly inflamed TILs and the last having no TILs (ID #8). Those not treated with immunotherapy but with TIL data (*n* = 3) had either no TILs or moderate TIL infiltration.

The average number of previous therapies prior to immunotherapy in the SWI/SNF-altered group was 1.7 (range, 1–4), with the overwhelming majority having been exposed to FOLFOX/FOLFIRINOX (*n* = 8; 89%) and 3 exposed to a gemcitabine regimen (33%). Other therapies tried before immunotherapy included capecitabine, cisplatin, and olaparib. In the 6 patients with SWI/SNF complex alterations not receiving immunotherapy, the average number of prior therapies was similar (1.8, range 1–3) to those in the SWI/SNF-altered patients who had immunotherapy (Table 1).

When immunotherapy was initiated, pembrolizumab was given most commonly. Other ICI drugs included durvalumab, nivolumab, and atezolizumab.

### Efficacy.

**Figure 4:**
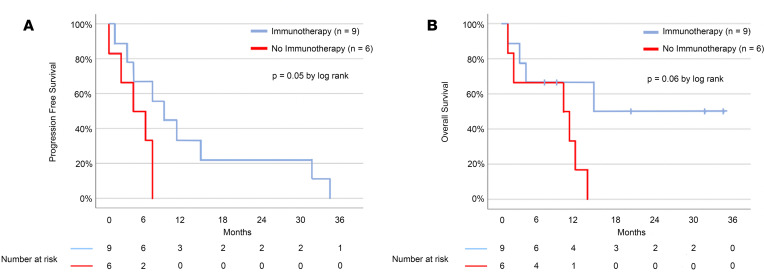
Kaplan-Meier estimates of progression-free survival and overall survival in patients with metastatic pancreatic ductal adenocarcinoma harboring SWI/SNF complex alterations treated with immunotherapy and without. (**A**) The median progression-free survival (PFS) was 9 months in the immunotherapy-treated pancreatic cancer patients with SWI/SNF alterations (blue line) versus 4 months in the patients not receiving immunotherapy (red line) (*P* = 0.05 by log rank). (**B**) The median overall survival (OS) was 15 months for the immunotherapy treated pancreatic cancer patients with SWI/SNF alterations (blue line) and 10 months for the patients not receiving immunotherapy (red line) (*P* = 0.06 by log rank). Tick marks indicate censored data.

For the 9 immunotherapy-treated SWI/SNF-altered metastatic pancreatic cancer patients, at date of data cut-off, median PFS was 9 months (95% CI, 3.1–14.8 months) and median overall survival (OS) was 15 months (95% CI, incalculable) (Figure 4). In comparison, nonimmunotherapy treated SWI/SNF*-*altered metastatic pancreatic cancer patients had a median PFS of 4 months (95% CI, 0–8.8 months) and a median OS of 10 months (95% CI, 0–20.8 months) (*P* = 0.05 and 0.06, respectively, for PFS and OS difference between immunotherapy-treated and nontreated SWI/SNF-altered pancreatic cancer patients). It should be kept in mind that the small number of patients precludes robust statistical analysis.

Altogether, 8 pancreatic cancer patients (89%) achieved an objective response in the SWI/SNF*-*altered, immunotherapy-treated population, with 1 achieving a complete response (CR; ID #1). In the nonimmunotherapy-treated SWI/SNF-altered treated group, no patients achieved an objective response.

Three patients with SWI/SNF-altered, immunotherapy-treated patients had dMMR disease (Supplemental Table 3, ID #1, #4, and #5); 2 of these patients achieved a partial response (PR), and 1 had a CR — their PFS was 15 months (for the CR), 36+, and 9+ months (for the PRs). In the 2 patients with dMMR disease in whom TMB was assessed, it was high: 58 and 23.8 mut/Mb. Patient ID #1 had *ARID1A*, *SMARCA4*, and *SMARCB1* alterations, while ID #4 had a *SMARCA4* alteration and ID #5 had an *ARID1A* and an *ARID1B* alteration. The remaining 6 patients’ tumors were MMR proficient. Of these patients, 5 achieved a PR and 1 was not evaluable for response (since they likely died of pneumonitis 1 month after starting therapy). The PFS in the patients attaining PR lasted 3, 4, 7+, 11, and 33+ months. The TMB in these patients ranged from 0 to 11 mut/Mb. These patients had alterations in *ARID1A* (ID #2, #7, #8, and #9), *PBRM1* (ID #3), and *ARID1B* and *SMARCA4* (ID #6).

Importantly, 4 pancreatic cancer patients in the database were treated with immunotherapy, even though they had no SWI/SNF alteration (Supplemental Table 2). The median PFS of the pancreatic cancer patients without SWI/SNF complex mutations who received immunotherapy was poor (median PFS = 2 months, OS = 9 months). The numbers are small, but these results are consistent with the literature.

### SWI/SNF alterations in pancreatic cancer do not correlate with a better prognosis.

By pooling 5 molecularly characterized pancreatic cancer gene data sets, we determined the prognostic impact of SWI/SNF complex alterations on OS (Supplemental Figure 1). There was no difference in survival between individuals with SWI/SNF-altered pancreatic cancer versus those with SWI/SNF WT pancreatic cancer.

## Discussion

Several studies have failed to show efficacy for ICI in pancreatic adenocarcinoma (39). It is postulated that the lack of responsiveness is due to a highly immunosuppressive tumor microenvironment, which makes it comparable with an immune-privileged site. In addition to PD-1 checkpoint inhibition, increased immunosuppressive cells such as Tregs, myeloid-derived suppressor cells (MDSCs), and tissue-associated macrophages (TAMs) restrict cytotoxic CD8^+^ and CD4^+^ T cell anticancer responses (15, 16, 40–43). Furthermore, most immunotherapy clinical trials that have been deployed in pancreatic cancer have not offered biomarker-based patient selection (15, 39).

Previously, several factors have been demonstrated to correlate with responsiveness to immunotherapy, including TMB ≥ 10 mut/Mb (11) and MSI-H (10). However, even with these tissue-agnostic biomarkers, there is a paucity of reports of these rare events and their correlation with immunotherapy outcomes in pancreatic cancer (13). Interestingly, in the pan-cancer setting, aberrations in chromatin remodeling genes of the SWI/SNF complex have also been shown to correlate with enhanced efficacy of ICI, although some of the data are inconsistent (27). For instance, *ARID1A* alterations are associated with better outcomes after immunotherapy across histologies; *PBRM1* alterations correlate with responsiveness to immunotherapy in some publications but not in others; and alterations in another chromatin remodeling gene — *SMARCA4* — are associated with responsiveness of small cell carcinoma of the ovary, hypercalcemic type (SCCOHT), to immunotherapy (27, 35, 44).

To our knowledge, this is the first study to evaluate immunotherapy in a group of patients with advanced pancreatic cancer who harbored alterations in chromatin remodeling genes. Pancreatic cancer genomic characterization through TCGA found SWI/SNF alterations in 10% of samples (*ARID1A*, 6%; *PBRM1*, 4%) (45). The International Cancer Genome Consortium (ICGC) data set found SWI/SNF alterations in 14% (46). Furthermore, we analyzed the aggregate SWI/SNF gene alterations within pancreatic cancer patients from 5 genomic data sets and found an alteration rate of 18% (Supplemental Figure 1) (46–48). Of our entire metastatic pancreatic cancer population with NGS available (123 patients), 15 had alterations in SWI/SNF complex members (12%), which is in line with the mutation rates of these large data sets.

Altogether, of our 9 treated patients, 8 (89%) achieved an objective response. The longest response is ongoing past 36 months, and the best response was a CR. Median PFS and OS were 9 and 15 months, respectively. As a comparison, objective response, median PFS, and OS with traditional second-line chemotherapy in pancreatic cancer is 17%, 3.1 months, and 6.1 months, respectively (49). Furthermore, first-line therapy in unselected metastatic pancreatic cancer has an average OS of approximately 11 months with either FOLFIRINOX or in contemporary gemcitabine plus nab-paclitaxel trials (5, 50, 51).

Importantly, overall, SWI/SNF complex alterations do not appear to be associated with significantly better prognostic outcomes from pooled genomic data sets (Supplemental Figure 1) (although individual SWI/SNF alterations might have prognostic significance, albeit with small numbers of patients assessed; ref. 52). As such, the ability of immunotherapy to improve outcomes in our SWI/SNF-altered metastatic pancreatic cancer patients likely has clinical relevance. However, there are characteristics of these SWI/SNF-altered pancreatic cancers that may be relevant. For instance, microsatellite instability was seen in 3 of the 12 SWI/SNF-altered pancreatic cancers with available data in the current report, while the established rate in pancreatic cancer overall is about 2.4% (53).

If the patients within this study were evaluated for the potential use of checkpoint inhibition based on current immune biomarkers, only 3 would have qualified, since they had dMMR. However, even in these types of patients, there is, up to now, a dearth of literature data specifically about the immunotherapy responsiveness of pancreatic cancer. These patients — ID #1, #4, and#5 (Supplemental Table 3) — had MSI-H, presumably caused by the mutations in MMR genes MSH2 (ID #1 and #5) and MSH6 (ID #4) per their molecular profile. Even so, epigenetic mechanisms such as hypermethylation can also contribute to MSI-H; in a proteomic screen, it was found that the ARID1A protein interacts with the MMR protein MSH2 and that ARID1A protein deficiency (as occurs when *ARID1A* is mutated as seen in patients ID #1 and #5) contributes to impaired MMR and a mutator phenotype (28). Of possible interest, tumors in 4 of our 9 immunotherapy patients (44%) did not harbor a *KRAS* mutation. Importantly, our SWI/SNF-altered pancreatic cancer group not receiving immunotherapy similarly did not have a *KRAS* mutation in 2 of 5 (40%) patients with available data, underscoring the likely genomic relevance of SWI/SNF complex members in pancreatic cancer carcinogenesis. Of note, however, cBioPortal data (https://www.cbioportal.org/) in pancreatic cancer patients with SWI/SNF-altered genes versus not showed that *KRAS* was mutated in the SWI/SNF-altered group at a rate of 85.1% (*n* = 160/188) versus SWI/SNF nonaltered harboring a *KRAS* alteration rate of 86.2% (*n* = 631/732) (54, 55). Given the fact that *KRAS* alterations are a hallmark driver mutation and occur in the vast majority of pancreatic cancers, whether or not the absence of *KRAS* mutations played a role in immune surveillance in our patients requires further investigation (56).

In terms of safety, 1 patient died from an immune-related adverse event (irAE) likely due to pneumonitis within 1 month of pembrolizumab initiation. This patient had previously been heavily pretreated (4 lines of therapy) and harbored pulmonary metastases. A second patient had a drug-induced myositis likely from the PD-L1 inhibitor atezolizumab at 17 months of treatment. Due to their ongoing PR, the multidisciplinary team thought it prudent to switch to the PD-1 inhibitor pembrolizumab, with ongoing good tolerance for an additional 16+ months (total PFS = 33+ months).

This study has several important limitations. First, the number of patients is small, and we did a retrospective analysis, which could be confounded by selection bias. Hence, prospective clinical trials are needed to validate the results, which should be considered preliminary. Furthermore, not all patients had complete immune profiling. Some patients received additional agents, along with their checkpoint inhibition. For instance, 1 patient received the MEK inhibitor trametinib with their anti-PD-1 agent; however, trametinib is not considered active in pancreatic cancer as a single agent. Another example is a patient who had previously failed FOLFIRINOX and gemcitabine plus nab-paclitaxel, who then received pembrolizumab together with gemcitabine plus nab-paclitaxel. Another confounder was that 3 of our 9 SWI/SNF-altered tumors were dMMR; however, our analysis shows that 5 of the 6 MMR-proficient, SWI/SNF-altered cancers achieved a PR, while no objective responses were seen in the 4 patients without SWI/SNF alterations who were treated with immunotherapy (and were MMR proficient). Also, as this was not a prospective study, it was not powered to detect survival differences between groups. Finally, a variety of laboratories provided the NGS and other immune studies; however, all laboratory tests were clinical grade.

In summary, the current analysis suggests that a subgroup of patients with pancreatic cancer and alterations in SWI/SNF complex chromatin remodeling genes, such as *ARID1A*, *ARID1B*, *PBRM1*, *SMARCA4*, and *SMARCB1*, can respond to ICI. Although one-third of these patients had MSI-H, the others had no MMR defect and only 3 had a TMB ≥ 10 mut/Mb. Furthermore, 5 of the 6 patients (83%) with low PD-L1 by IHC achieved an objective response. Several studies have previously shown that genomic profiling can assist with patient selection for a variety of therapies (27, 57, 58). Taken together, our current data suggest that prospective studies of ICI are warranted in patients with advanced pancreatic cancer whose tumors harbor alterations in chromatin remodeling genes.

## Methods

### Study population and approval.

Patient data was curated from the electronic medical records. The Profile Related Evidence Determining Individualized Cancer Therapy (PREDICT, NCT02478931) database of eligible patients at the Center for Personalized Cancer Therapy (UCSD Moores Cancer Center), whose tissue DNA was analyzed by NGS was searched for patients who had clinically staged metastatic and histologically confirmed pancreatic ductal adenocarcinoma only, had completed NGS, and were treated with checkpoint inhibitor immunotherapy. The cBio Cancer Genomics Portal was analyzed for 5 additional pancreatic adenocarcinoma gene data sets. For survival analysis across all pancreatic cancer patients from the 5 available data sets (59–63), we stratified by SWI/SNF complex alteration and evaluated OS.

### Molecular testing and other biologic markers.

NGS of tissue DNA and/or blood ctDNA was performed in clinical laboratory improvement amendment (CLIA) laboratories, including, most commonly, Foundation Medicine (foundationmedicine.com), Tempus (tempus.com), and UCSD for tissue DNA (NGS panel sizes from > 180 to > 400 genes); and Guardant (guardanthealth.com; panel size ~70 genes) — the latter most commonly for ctDNA NGS. Other platforms for NGS testing included Caris (carismolecularintelligence.com), Omniseq (omniseq.com), Paradigm (paradigmdx.com), Nanthealth (nanthealth.com), and Pathline (pathlinelabs.com).

Patients had their NGS report evaluated for alterations in SWI/SNF complex members, focusing on *ARID* and *SMARC* family genes, as well as *PBRM1* (Supplemental Table 1). Only characterized SWI/SNF complex members were evaluated; all variants of unknown significance were excluded from further study.

MMR protein proficiency was determined by expression of MLH1, MSH2, PMS2, and MSH6 IHC staining by CLIA-licensed laboratories as specified above. TMB was determined either by subtracting germline from somatic tumor sequencing when available or by computational analysis when only tumor sequencing was available. Although gathered from multiple labs and chronologic specimens, TMB were all standardized and expressed as the number of mut/Mb. All TMB analysis was prior to ICB therapy and stratified as: low (<6 mut/Mb), intermediate (6–19 mut/Mb), or high (>19 mut/Mb) (11). Immune profiling was done per laboratory specification and at a minimum included PD-L1 determination by IHC, while more expansive panels evaluated PD-1, TILs, and the quantity of infiltrating CD8+ T cells. PD-L1 expression was stratified on the following scale: low (0%–1%), intermediate (2%–49%), and high (50%+) using the Dako 22C3 pharmDx qualitative immunohistochemical assay (38).

### Statistics.

PFS and OS were measured from the first date of immunotherapy until the cut-off date of 6/1/2020 (or the last time of contact). These were plotted by the Kaplan-Meier method, and any patients who did not progress or were alive at the date of data cut-off (or time of last contact) were censored for PFS or OS, respectively, at that time point. Responses (PR and CR) were evaluated by RECIST assessment per physician. Statistical analysis was completed using the SPSS software package.

### Study approval.

This study was performed in accordance with UCSD IRB guidelines for data analysis and for any investigational treatments for which patients gave consent.

## Author contributions

GPB, SK, HP, PF, SL, and RO collected patient data. GPB, SK, and RK conceived, analyzed, and wrote the manuscript. All authors edited and approved the manuscript.

## Supplementary Material

Supplemental data

Trial reporting checklists

ICMJE disclosure forms

## Figures and Tables

**Table 1:**
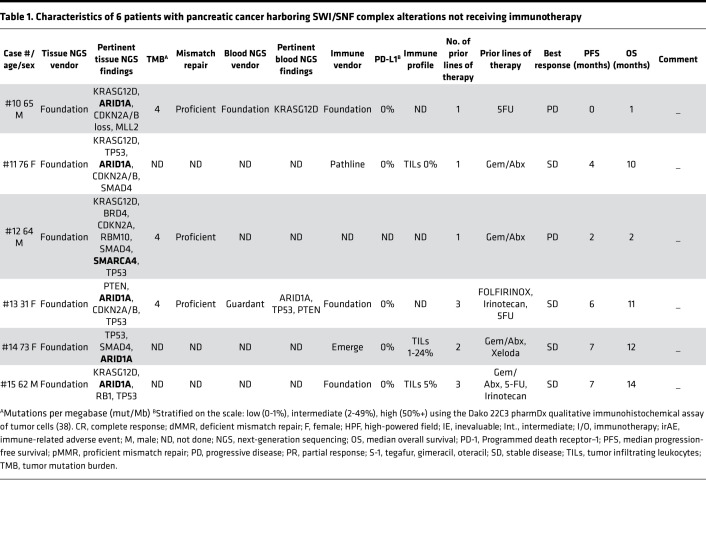
Characteristics of 6 patients with pancreatic cancer harboring SWI/SNF complex alterations not receiving immunotherapy

## Version 1 08/10/2021

In-Press Preview

## Version 2 09/22/2021

Electronic publication
