# Supplementary material for: SWI/SNF complex alterations as a biomarker of immunotherapy efficacy in pancreatic cancer
Source: JCI Insight. 2021 Sep 22;6(18):e150453. doi: 10.1172/jci.insight.150453 (PMC8492298; doi:10.1172/jci.insight.150453)
Supplement: Supplemental data [file jciinsight-6-150453-s258.pdf]

Supplemental Table 1. Next Generation Sequencing of SWI/SNF Sub-Unit Genes

Family	Gene Name	Protein Name
ARID		
	ARID1A	ARID1A
	ARID1B	ARID1B
	ARID2	ARID2
SMARC		
	SMARCA2	SMARCA2 / Probable global transcription activator SNF2L2
	SMARCA4	SMARCA4 / Transcription activator BRG1
	SMARCB1	SMARCB1
	SMARCC1	SMARCC1
	SMARCC2	SMARCC2
	SMARCD1	SMARCD1
	SMARCD2	SMARCD2
	SMARCD3	SMARCD3
	SMARCE1	SMARCE1
PBRM		
	PBRM1	PRBM1

Supplemental Table 2: Characteristics of Four Patients with Pancreatic Cancer Not Harboring SWI/SNF Complex Alterations Receiving Immunotherapy

Case # / Age/ Sex	Tissue NGS Vendor	Pertinent Tissue NGS Findings	TMB*	Mismatch Repair	Blood NGS Vendor	Pertinent Blood NGS Findings	Immune Vendor	PD-L1**	Immune Profile	# Prior Lines of Therapy	Prior Lines of Therapy	Immunotherapy	Best Response	PFS (months)	OS (months)	Comment
#16 62 / M	Foundation	KRAS G12V, CDKN2A/B loss exons 2-3, TP53 G244C	5	Proficient	Guardant	APC H250D	Foundation	0%	TILs 0%, PD-1 10%	2	gemcitabine + nab-paclitaxel	Pembrolizumab	PD	2	13	After immunotherapy progression, patient started on 5-FU + liposomal irinotecan followed by gemcitabine alone
#17 64 / M	Foundation	NRAS amplification, CDKN2A p16INK4a G6_A21del, TP53 S215R	ND	Proficient	Guardant	TP53 S215R, MYC amplification, CDKN2A G6_A21del	Caris	0%	PD-1 0%	1	gemcitabine + nab-paclitaxel + cisplatin	oleclumab + Durvalumab	PD	1	9	After immunotherapy progression patient started FOLFOX until death
	Caris	HER2 negative, TS Positive, TUBB3 Positive	ND	Proficient												
#18 75 / F	Foundation	KRAS G12D, CDKN2A p16INK4a R29_A34del, TP53 G199V	6	Proficient	ND	ND	Omniseq	0%	TILs Low, CD8+ non-infiltrating, PD-L1/L2 copy number not amplified	1	Gemcitabine	pembrolizumab	PD	2	3	–
	Tempus	TP53 G199V, KRAS G12D	4.2	Proficient			Tempus	0%								
#19 60 / M	Foundation	KRAS G12V, CDKN2A/B loss, SMAD4 loss, TP53 P278S	3.51	Proficient	ND	ND	ND	ND	ND	0	Nothing	pembrolizumab + gemcitabine + nab-paclitaxel + IDO-1	PD	4	9	After immunotherapy progression patient started on 5FU + liposomal irinotecan + IL-alpha inhibitor

* = mutations per megabase (mut/Mb)

** = stratified on the scale: low (0-1%), intermediate (2-49%), high (50%+) using the Dako 22C3 pharmDx qualitative immunohistochemical assay of tumor cells[38].

*** = "+" means ongoing response

Abbreviations: CR = Complete Response; dMMR = deficient mismatch repair; F = female; HPF = high powered field; IE = Inevaluable; Int. = Intermediate; I/O = Immunotherapy; irAE = immune related adverse event; M = male; ND = Not done; NGS = next generation sequencing; OS = median overall survival; PD-1 = Programmed death receptor–1; PFS = Median Progression Free Survival; pMMR = proficient mismatch repair; PR = partial response; S-1 = tegafur, gimeracil, oteracil; SD = stable disease; TILs = tumor infiltrating leukocytes; TMB = tumor mutation burden

Supplemental Table 3: Characteristics of Nine Patients with Pancreatic Cancer Harboring SWI/SNF Complex Alterations Receiving Immunotherapy

Case # / Age/ Sex	Tissue NGS Vendor	Pertinent Tissue NGS Findings	TMB *	Mismatch Repair	Blood NGS Vendor	Pertinent Blood NGS Findings	Immune Vendor	PD-L1 **	Immune Profile	# Prior Lines of Therapy	Prior Lines of Therapy	Immuno-therapy	Best Response	PFS (months)	OS (months)	Comment
#1 79 M	Caris	**ARID1A** G276fs, FBXW2 R465H, FGFR3 R248C, KMT2D R2830X, PIK3CA E81K, **SMARCA4** T910M, **SMARCB1** R377C, ATM Exon 7, ATM Exon 10, ATM Exon 63, FGFR3 Exon 7, TSC1 Exon 15 P603fs, MSH2 R383X	58	Deficient	ND	ND	Caris	Low	ND	1	FOLFIRINOX	Pembrolizumab	CR	15	15	_
#2 60 F	Paradigm	**ARID1A** E2033, CCNE1 Gain, KRAS G12V, TP53 R156P	8	Proficient	Guardant	TP53 R156P, KRAS G12V	Paradigm	Low	ND	2	FOLFOX gemcitabine + nab-paclitaxel	Pembrolizumab + Trametinib	PR	4	4	–
	Tempus	**ARID1A** E2250, TP53 R156P, KRAS G12V, CDKN2A copy number loss	3.3	Proficient	Tempus	KRAS G12V, TP53										
#3 47 F	Caris	CREBBPP Exon 16 E1058fs, **PBRM1** Exon 12 Y417fs, VTCN1/	7	Proficient	Guardant	CDK6 F164F	Caris	Low	TILs moderately inflamed PD-1 Very High	2	FOLFIRINOX Gemcitabine + nab-paclitaxel	Pembrolizumab plus nab-paclitaxel + gemcitabine	PR	11	21+***	–
	Ashion	NRG1 Fusion	2	Proficient			Omniseq	Low								
#4 70 F	Foundation	**SMARCA4** R1135W, ERBB3 G284R, CDKN2A R80, DNMT3A G543A, TP53 R273C, KRAS G12V, CDKN2A R58, FBXW7 R505C, MSH6 2521delA	ND	Deficient	ND	ND	Omniseq	Int.	TILs 10% - 20%	1	FOLFIRINOX	Durvalumab based therapy	PR	36+	36+	_
#5 68 F	Tempus	**ARID1A** M835fs, KRAS G12D, MEN1 R516fs, KMT2C K2797fs, NBN R466fs, FAT1 F3952fs, POT1 39-2A>G, KMT2D P647fs, SLIT2 N155fs, KMT2D E5011, SMAD2 R427, **ARID1B R1944,** RPL5 D59fs, RAD50 E723fs, MSH2 N84fs	23.8	Deficient	Guardant	Negative	ND	ND	ND	1	FOLFIRINOX	Pembrolizumab	PR	9+	9+	_

Case # / Age/ Sex	Tissue NGS Vendor	Pertinent Tissue NGS Findings	TMB *	Mismatch Repair	Blood NGS Vendor	Pertinent Blood NGS Findings	Immune Vendor	PD-L1 **	Immune Profile	# Prior Lines of Therapy	Prior Lines of Therapy	Immuno-therapy	Best Response	PFS (months)	OS (months)	Comment
#6 66 M	Foundation	**ARID1B** A757T, **SMARCA4** S312Y, ALK V564M, IRS2 N28_H29insN, MAGI2 E867K, MPL I492M, NOTCH1 G269R, TSC2 A460T, ERBB3 G337R, BRCA2 S1982fs, FBXW7 L700fs, AXIN 1 R533_H534insQVHH, BCORL1 splice site 4531_4618+102del190, EP300 E1492, KEP splice site 1532-2A>T	11	Proficient	ND	ND	Caris	Int.	PD-1 >5/HPF	1	FOLFOX	Atezolizumab followed by pembrolizumab	PR	33+	33+	Switched from atezolizumab to pembrolizumab in setting of drug induced myositis
							Paradigm									
	Tempus	BRCA2 51982fs, KEAP 1532-2A>T, EP300 E1492, NBN 5615fs, BCORL1 Y1585fsPD-L1 5%	8.3	Proficient			Tempus		ND							
#7 60 M	Foundation	**ARID1A** Q2039fs, EZR-ERBB4 fusion (E10-E18), PIK3CA R88Q	1	Proficient	Tempus	Negative	Omniseq	Low	TILs Highly Inflamed CD8 Moderately Infiltrating	1	FOLFIRINOX	Nivolumab plus gemcitabine-based therapy	PR	7+	7+	–
#8 67 F	Paradigm	KRAS G12D, ERBB2, MGMT, TOP1,	7	Proficient	Guardant	Negative	Emerge	Low	TILs Negative PD-1 Negative	4	Gemcitabine	Pembrolizumab	IE	1	1	Patient died secondary to irAE pneumonitis one month after starting I/O
	Foundation	**ARID1A** L2064fs, TP53 W53, KRAS G12D, ERBB2 R678Q			Guardant	KIT H894H					Capecitabine + radiation					
	Foundation	**ARID1A** L2064fs, TP53 W53, KRAS G12D, ERBB2 R678Q, BRCA1 truncation intron 16, CDKN1B G97fs, CDKN2A/B loss			Guardant	TP53 W53, CCNE1 K209Q, KIT H894H					Gemcitabine + Cisplatin					
											Cisplatin + Olaparib					
#9 67 F	Foundation	**ARID1A** H1843fs, BRCA1 truncation intron 16, KRAS G12V, CDKN2A/B loss, SMAD2 intron 5, TP53 T155N	0	Proficient	Guardant	KRAS G12V, TP53 T155N, **ARID1A H1843fs**	Omniseq	Low	CD8 Minimally Infiltrating, TMB 4.3 mut/Mb, pMMR	3	S-1	Pembrolizumab	PR	3	3	–
					Foundation	KRAS G12V, TP53 T155N					Olaparib					
					Guardant	KRAS G12V, TP53 T155N, ARID1A H1843fs, TP53 T256P, TP53 L265P					FOLFIRINOX					

* = mutations per megabase (mut/Mb)
** = stratified on the scale: low (0-1%), intermediate (2-49%), high (50%+) using the Dako 22C3 pharmDx qualitative immunohistochemical assay of tumor cells(38).
*** = "+" means ongoing response
Abbreviations: CR = Complete Response; dMMR = deficient mismatch repair; F = female; HPF = high powered field; IE = Inevaluable; Int. = Intermediate; I/O = Immunotherapy; irAE = immune related adverse event; M = male; ND = Not done; NGS = next generation sequencing; OS = median overall survival; PD-1 = Programmed death receptor–1; PFS = Median Progression Free Survival; pMMR = proficient mismatch repair; PR = partial response; S-1 = tegafur, gimeracil, oteracil; SD = stable disease; TILs = tumor infiltrating leukocytes; TMB = tumor mutation burden

Supplemental Figure 1. Overall Survival of SWI/SNF Complex Altered versus Wild-type in Pancreatic Cancer.

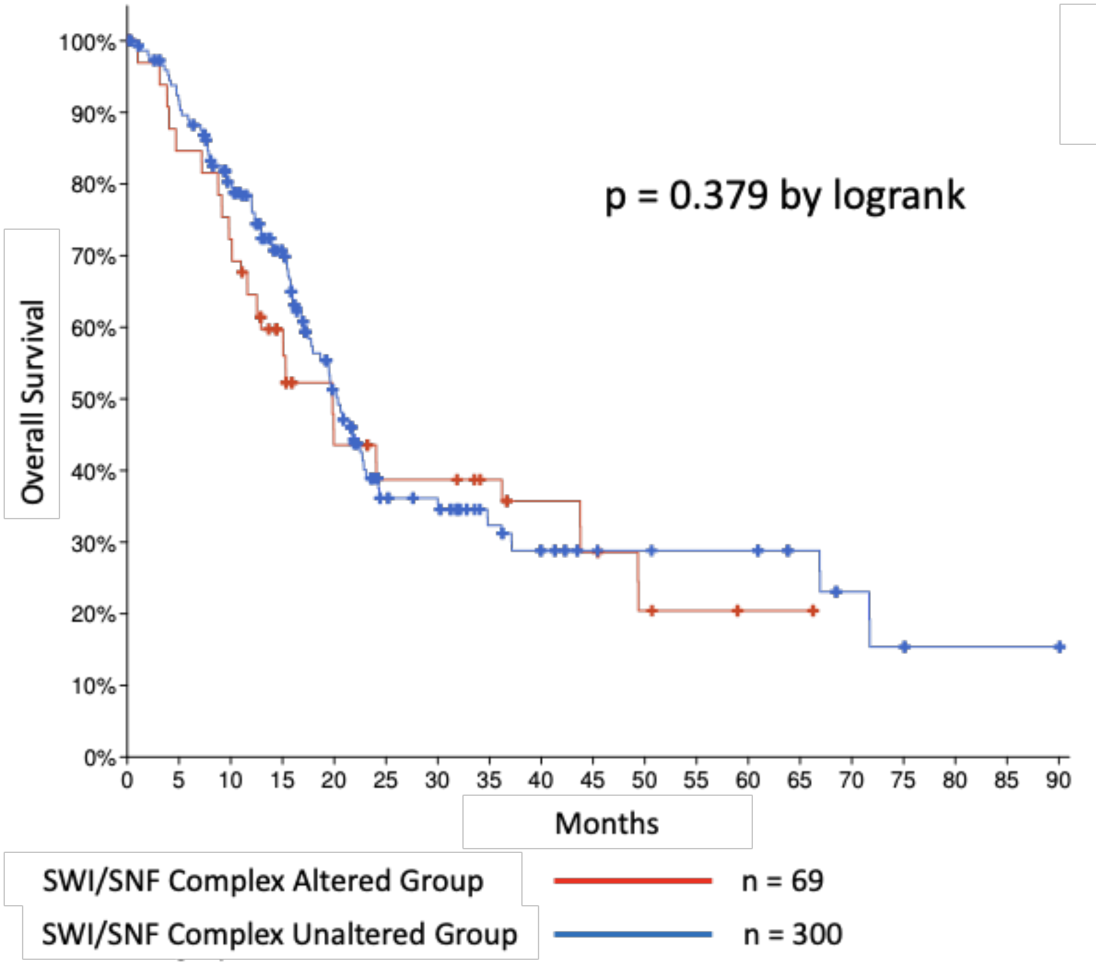

Supplemental Figure 1. Overall Survival of SWI/SNF Complex Altered versus Wild-type in Pancreatic Cancer.
The cBioPortal for Cancer Genomics was used to query patients from 5 pancreatic carcinoma genomic datasets (ICGC, Nature 2012; QCMG, Nature 2016; TCGA, Firehose Legacy; TCGA, PanCancer Atlas; UTSW, Nat Commun 2015). Red line = altered SWI/SNF complex gene as specified. Blue line = unaltered SWI/SNF complex gene. Genes examined include *ARID1A, ARID1B, ARID2, SMARCA2, SMARCA4, SMARCB1, SMARCC1, SMARCC2, SMARCD1-3, SMARCE1,* and *PBRM1.* SWI/SNF-altered versus wild-type pancreatic cancer had no difference in survival (p= 0.379).
